# Anti-Inflammatory Peptide Prevents Aβ_25–35_-Induced Inflammation in Rats via Lipoxygenase Inhibition

**DOI:** 10.3390/cells14130957

**Published:** 2025-06-23

**Authors:** Yudhishthir Yadav, Masroor Anwar, Hanuman Sharma, Suman Jain, Uma Sharma, Partha Haldar, Aparajit B. Dey, Sharmistha Dey

**Affiliations:** 1 Department of Biophysics, All India Institute of Medical Sciences, New Delhi 110029, India; yudhishthir.yadav7@gmail.com (Y.Y.); masroor_anwar07@yahoo.com (M.A.); 2 Bioanalytics Facility, All India Institute of Medical Sciences, New Delhi 110029, India; hpsharma.aiims@gmail.com; 3 Department of Physiology, All India Institute of Medical Sciences, New Delhi 110029, India; sumanjain10@gmail.com; 4 Department Nuclear Magnetic Resonance, All India Institute of Medical Sciences, New Delhi 110029, India; umasharma69@gmail.com; 5 Department of Community Medicine, All India Institute of Medical Sciences, New Delhi 110029, India; parthahaldar@outlook.com; 6 Department of Geriatric Medicine, All India Institute of Medical Sciences, New Delhi 110029, India; abdey@hotmail.com

**Keywords:** lipoxygenase, cognitive behavioral assessment, MRI scan, AD rat model, immunoblot, immunofluorescence

## Abstract

Neuroinflammation, triggered by lipoxygenase (LOX), contributes to Alzheimer’s disease (AD) progression. Overexpression of LOX-5 in patients with AD serum highlights its role. This study assessed the efficacy of the LOX-inhibitor-peptide YWCS in an AD rat model induced by Aβ_25–35_ injection. Cognitive tests, magnetic resonance imaging (MRI) scans, and molecular analyses were conducted. YWCS treatment significantly improved cognitive function, as evidenced by improved performance in the open field, novel object recognition, elevated plus maze, and Morris water maze tests. MRI scans revealed hippocampal shrinkage in AD rats and no changes were observed from YWCS treatment. Molecular analysis revealed altered expression of LOX-5, LOX-12, Aβ, γ-secretase components, p-Tau^181^, Akt, p-Akt, and p53 in AD rats. Immunofluorescence staining confirmed increased expression of LOX, Aβ, and p-Tau^181^ in the hippocampus of AD rats, which was reduced by YWCS treatment. Serum LOX levels were elevated in AD rats and significantly decreased after YWCS treatment, aligning with previous findings in human AD patients and AD cell models. YWCS offered improvements in behavioral and inflammatory marker regulation and also prevented progression of the disease, as shown by MRI results. These results suggest that YWCS, by targeting LOX, has the potential to be a promising therapeutic agent for AD.

## 1. Introduction

The growing number of older individuals and the current lack of effective treatments for Alzheimer’s disease (AD), the leading cause of dementia, has become a major global health crisis with significant social and economic load. The primary pathological indicators of AD include the presence of amyloid plaques, largely composed of neurotoxic Aβ-amyloid peptides, and intracellular neurofibrillary tangles, which are mainly formed by hyperphosphorylated tau. Research has suggested that neuroinflammation is a key feature of the AD brain. Injury to the head followed by inflammation of the brain is a risk factor for AD. The attack by microglia to remove toxic Aβ-amyloid produces large amounts of inflammatory cytokines which fail to remove persistent amyloid deposits and produce damage to the brain. Inflammation and toxic insults to the aging brain also help the progression of AD pathology and the neuroinflammation mediated by lipoxygenase (LOX) [1,2,3]. In AD, upregulation of lipoxygenase-5 (LOX-5), a pro-inflammatory enzyme with widespread presence in the central nervous system, has been observed. Other isoforms of LOX (LOX-12/15) were found to be overexpressed in cerebrospinal fluid of AD and mild cognitive impairment (MCI) [4]. Overexpression of LOX-5 causes synaptic failure, as found by the loss of synaptic biomarkers in a murine AD model [5,6]. LOX-5 contributes to Aβ formation by regulating the γ-secretase complex and impacts tau metabolism by modifying its phosphorylation state at specific epitopes through cdk-5. In vivo investigations have revealed that suppressing or eliminating LOX-5 substantially lowers γ-secretase expression and Aβ levels [7] and decreases tau phosphorylation [8]. The specific LOX inhibitor zileuton attenuates ischemic brain damage by inhibiting inflammatory reactions through the activation of the PI3K/Akt signaling pathway [9]. Hence, the LOX-5 pathway appears to be significantly involved in the complete development of AD pathology, which includes aberrant Aβ production and deposition as well as altered tau phosphorylation [10]. Therefore, targeting LOX-5 may bridge the current AD treatment gap by being both a therapeutic as well as a preventative molecule. Based on previous studies, specific inhibition of LOX-5/12 may represent a significant promising avenue for AD patient therapy. In our previous study, LOX-5 was found to be significantly overexpressed in the serum of AD patients compared to a normal control group, hence LOX-5 is a possible serum protein biomarker for AD. The same study also provided information regarding the potent LOX-5 peptide inhibitor YWCS, which prevents the neurotoxic impact of Aβ_25–35_ by involving a mechanism that reduces the expression of γ-secretase and levels of p-Tau^181^ in SHSY5Y neuroblastoma cells [11]. Because of the structural similarities of LOX-5 and LOX-12 [12], this peptide (YWCS) functions as an inhibitor of both LOX isoforms and also acts as a dual inhibitor in breast cancer patients as well as in AD sufferers. Peptide YWCS presents a potential novel avenue for inhibiting LOX-5, which could offer a protective strategy against neurotoxicity associated with AD. Aβ_25–35_ can form aggregates similar to the plaques observed in AD patients, formed by proteolytic cleavage of soluble, full-length Aβ peptides, and has been found to be a major component of these plaques [13]. Aβ_25–35_ is characterized as a biologically active region of Aβ as it is the shortest fragment that exhibits large β-sheet aggregated structures and retains the toxicity of the full-length peptide [14,15,16,17]. The AD rat model, developed by i.c.v. administration of Aβ_25–35_ peptide in the brain, exhibits neurotoxic effects and aggregation properties similar to the full-length peptide [18,19,20,21]. Furthermore, studies have shown that rats treated with Aβ_25–35_ had increased mRNA levels of the amyloid precursor protein APP [22]. This suggests that Aβ_25–35_ treatment can induce a change in the expression of APP, a key protein involved in the production of amyloid beta (Aβ) peptides, including Aβ_25–35_ itself. The study reported Aβ_25–35_ induced apoptosis and tau hyperphosphorylation by regulating PI3K/Akt/GSK-3β signaling [23]. These research findings provide a valuable tool for developing therapeutic molecules for Alzheimer’s disease [24].

In light of the above evidence, the current study evaluated the efficacy of a peptide inhibitor of LOX in a rat model of AD to translate our in vitro results. The AD rat model offers valuable tools for evaluating new therapeutic strategies for the treatment of human diseases, as well as for studying the pathological mechanisms involved in diseases. One of the critical features of the AD animal model is the ability to analyze memory and cognition status using behavioral tests. This study will help in creating an AD rat model and help to accumulate all possible behavioral data and brain MRIs and alterations from the effects of the peptide inhibitor of LOX. It will further explore the changes at the molecular level affected in AD by targeting LOX.

## 2. Materials and Methods

### 2.1. Synthesis of Peptides—Aβ_25–35_ and YWCS

The peptide YWCS (an inhibitor of LOX-5 and LOX-12) was designed by molecular modeling, as reported in our previous paper [12]. Both the peptides Aβ_25–35_ and YWCS were synthesized by solid phase peptide synthesis (SPSS, latest v. 30) by using Fmoc and Wang resin chemistry, as described previously [25].

### 2.2. Mass Spectrometric Analysis of Aβ_25–35_ and YWCS Peptides

The mass spectrometric analysis of peptides Aβ_25–35_ and YWCS was performed using a QTRAP 6500+ (Sciex, Framingham, MA, USA). The parameters of the mass spectrometer were controlled using Analyst (ver 1.7.1; Sciex, Framingham, MA, USA).

### 2.3. Experimental Rats and Groups

The experiments were performed in accordance with the guidelines laid by the Committee for the Purpose of Control and Supervision of Experiments on Animals (CPCSEA), Govt. of India. The experimental protocol of this study was approved by the Institutional Animal Ethics Committee (IAEC) of the All India Institute of Medical Sciences, New Delhi (File No: 145/IAEC-1/2019, Approval Date: 4 May 2019). All efforts were made to minimize the number of animals used and their pain during surgery and postoperative recovery. Male Wistar rats weighing between 250 ± 20 g which were bred, reared, and maintained in the Central Animal Facility of the All India Institute of Medical Sciences (AIIMS), New Delhi, India, were used for the experiments. The animals were housed in individual polypropylene cages in an animal room with controlled room temperature (25 ± 1 °C), having 12 h light/dark cycle at room and normal humidity. After two weeks of acclimatization, the animals were randomly assigned and food and water were freely available to the rats. The rats were randomly divided into five groups (*n* = 6 rats per group). All the groups of rats were left for an incubation period of 60 days. The five different groups are: (1) normal control (no surgery/treatment was given); (2) sham (0.9% PBS administered ICV); (3) Aβ_25–35_ (Aβ_25–35_ 30 µg, bilaterally, administered intracerebroventricularly); (4) Aβ_25–35_ (30 µg ICV) + peptide YWCS 5.0 mg/kg (administered intranasally); (5) Aβ_25–35_ (30 µg ICV) + peptide YWCS 10 mg/kg (administered intranasally).

### 2.4. Intracerebroventricular (i.c.v.) Injection of Aggregated Aβ_25–35_ Peptide

The Aβ_25–35_ peptide is considered the biologically active fragment of full-length Aβ_1–42_ because it is the shortest Aβ peptide that retains full toxicity [26].

The synthesized Aβ_25–35_ peptide was reconstituted by dissolving in 200 µL of phosphate-buffered saline at the concentration of 1 μg/μL and the solution was incubated at 37 °C for Aβ_25–35_ aggregation for 1 week before use. Under anesthesia (a combination of 0.05 mL/100 g body weight of xylazine and 0.1 mL/100 g bodyweight of ketamine) a rat was injected with aggregated Aβ_25–35_. The head was fixed onto the stereotaxic platform (Stereotaxic Injector, Stoelting Co., Wood Dale, IL, USA) by securing the ear and snout knobs, followed by a median sagittal incision after the removal of hairs around the skull region. The Bregma, which is a juncture of sagittal and coronal suture, was marked as a reference point and two holes were drilled on either side over the lateral ventricles. The aggregated Aβ_25–35_ (30 µg) and PBS were injected at a rate of 1 µL/ min for the AD rat group and sham group, respectively. To each lateral ventricle, 15 µg of aggregated Aβ_25–35_ was administered bilaterally (i.c.v.) using a Hamilton micro-syringe [−3.5 mm dorsoventral (DV), 0.84 mm anteroposterior (AP), and 1.5 mm mediolateral (ML) to the Bregma]. The injector was left in the injection site for 5 min to allow complete diffusion of aggregated Aβ_25–35_ peptide. Utmost care was taken to avoid any chance of excess blood loss. After sealing the incision with surgical thread, for post-surgery care, the rat was kept on a heating pad and each rat was caged individually and was observed carefully until full consciousness was regained. A post-surgery antibiotic was given intramuscularly, 10 mg/kg, at hindlimbs of the rats, and food was allocated inside the cage for a few days (4–5 days).

### 2.5. Intranasal Administration of the Peptide YWCS

The peptide YWCS was administered twice intranasally in both the nostrils of the rats daily for a period of 30 days. After the completion of the treatment duration, the rats were assessed for neurobehavioral and molecular parameters.

### 2.6. Behavior Study

The behavioral tests were performed in a soundproof room during the daytime. Tests were recorded with the aid of a camera fixed on top of the behavioral apparatus, followed by its analysis through ANY-maze software, 6.33 (Stoelting Co., Wood Dale, IL, USA). The open field (OF), elevated plus maze (EPM), novel object recognition (NOR) and Morris water maze (MWM) behavioral tests were performed in all the groups at the following three points: at baseline, after Aβ_25–35_ ICV injection, and after peptide YWCS intranasal treatment:

#### 2.6.1. Open Field (OF)

The open field test is the most widely used behavioral setup which measures the psychology of the animal. It is a very rapid and easy method for the analysis of the emotionality of the animal. In this setup, a rat is kept in the middle of an empty, walled enclosed area with its head facing towards one side of the cage wall. Locomotor activity was recorded during the experiment. The duration of movement and time spent in the center were analyzed using the computerized video tracking system Anymaze (Stoelting Co., USA), and the rat was left to explore for 5 min. A track plot showed the movement of the rats in an open arena.

#### 2.6.2. Elevated Plus Maze (EPM)

The elevated plus maze (EPM) is a well-known behavioral experiment that is used to access the anxiety response of rodents. The anxiety behavior is accessed by the ratio of time duration spent in the open arm to the time spent in the closed arm. The EPM was kept at an elevation of 50 cm from the floor. Indirect, less intensity light exposure was kept during the experiment to match with the dark phase of the circadian cycle of the rat. A video recorder was placed above the maze to capture the movement and activity of the rat. The activity includes the time spent in the open arm and closed arm, and the track plot shows the path chosen by the rats in the open arm and closed arm.

#### 2.6.3. Novel Object Recognition (NOR)

The novel object recognition test is a cognitive analysis test which was performed to test the recognition memory of the rodent animal. It evaluated the animal’s ability to differentiate and recognize a novel object from a known object based on the recognition index (RI). The NOR test consisted of the following three consecutive phases: habituation, familiarization, and the actual test phase. The primary objective of the habituation phase was to acclimatize the rats to the testing environment itself. The habituation stage is known as the T0 stage and is followed by the T1 stage, i.e., the training stage. The training stage was performed 24 h after the completion of the T0 stage. In the training stage, two objects of consistent height and volume were kept diagonally in the cage (familiar arena) and the rat was allowed to explore the open field for 5 min in the presence of the same objects so to familiarize the rat with them. After T1, the rats were kept individually in a cage to prevent alteration in the behavior of other rats due to a change in behavior of the trained rat. After 20 min, the rats were set for the test stage, i.e., T2. In the test stage, the rats were allowed to explore the open field in the presence of a familiar object and one novel object of a different shape and texture for 5 min. The behavior of the rats were recorded and quantitatively evaluated to uncover their preferences and memory performance. The recognition index (RI) was calculated.

#### 2.6.4. Morris Water Maze (MWM)

The spatial memory and learning of rats were evaluated using the Morris water maze test. They were trained to allocate a hidden platform in reference to visual cues. This task consisted of the following three stages: place navigation, cued navigation, and probe test. The MWM task was conducted using a circular pool filled with water to a depth of 30 cm. The pool was divided into four equal quadrants with visual cues of different colors and shapes pasted in each quadrant. A transparent circular platform was positioned below the water surface at the center of one of the quadrants. The position of the platform was not disturbed during the four days of the training period. The MWM task was completed over 7 consecutive days. For the first six days, the rat was trained to find the position of a fixed, hidden platform. The rat was placed into the pool facing the wall and allowed to search for the hidden platform. After completion of the place navigation task during days 1–6, the platform was removed for the probe test on day 7. The time spent in the target quadrant where the hidden platform was placed previously was assessed as an index of spatial memory. Also, the track plot indicated the path chosen by each rat to reach the target quadrant. A shorter path to reach the target quadrant, more time spent in the target quadrant, and the latency to reach the platform in the target zone were chosen as indicative parameters for good spatial memory.

### 2.7. MRI of Brain Volumetric Analysis

An MRI test of each rat was performed thrice, once at baseline, once after injection of aggregated Aβ_25–35_ (AD rats) through ICV, and once after intranasal treatment with peptide YWCS. MRI brain imaging was performed with a Bruker BioSpin MRI GmbHBioSpec 70/20US Avance III 7T system, equipped with a transmit cylindrical radiofrequency coil (8.6 cm inner diameter) and a rat brain dedicated receive-only array coil (2 × 2 elements) positioned over the rat’s head. The rats were anesthetized with isoflurane (2.0% and 1.5–2.0%). The oxygen was supplied during the MRI session, and its head was secured in a head holder and placed on a dedicated rat scan bed in the prone position with plastic retainers for the teeth and ears (Bruker, BioSpin, Billerica, MA, USA). The rat was wrapped with a flexible textile to prevent limb movements during the scanning process. The correct positioning of each animal within the RF coil was confirmed through a series of scout images. The spin-echo coronal images were acquired (T2 TurboRARE, 4:1) using the following parameters: repetition time (TR) = 2500 ms, echo-time (TE) = 33 ms, scan = 4, field of view (FOV) = 3.50 cm, Pos = 2.90 mmD, FA = 90.0 deg, number of averages = 6, scan time = 8 min and 5 sec, and twenty 0.5 mm-thick slices. The spin-echo axial images were acquired (T2-TurboRARE, 3:1) using the following parameters: repetition time (TR) = 2639.3 ms, echo-time (TE) = 33 ms, scan = 3, field of view (FOV) = 3.50 cm, Pos = 4.76 mm Cd, FA 90.0 deg, number of averages = 6, scan time = 8 min and 17 sec, and twenty-five 0.5 mm-thick slices. MRI images were exported in DICOM format (.dcm) and then converted to NifTI format (.nii). Physiological parameters, i.e., respiration rate and body temperature, were monitored throughout the imaging sessions.

### 2.8. Isolation of Brain Tissue and Processing

#### Tissue Isolation and Lysate Preparation

The frontal cortex and hippocampus are usually the primary brain region affected in AD and play a fundamental role in memory and learning. After completion of the treatments, the rats were anesthetized and sacrificed, and the brains were removed carefully: the frontal cortex (FC) and hippocampal (HC) tissue were isolated. The tissue was homogenized in a tissue dissociator (Miltenyi Biotec, Bergisch Gladbach, Germany) in RIPA buffer (GBiosciences, St. Louis, MO, USA), followed by centrifugation at a speed of 13,000 rpm for 20 min at 4 °C. The supernatant was collected and stored at −80 °C to avoid denaturation of the proteins for further experiments.

### 2.9. Immunoblotting

Total protein concentration was determined by using a Bicinchoninic acid assay (BCA). The proteins (30–50 μg) were electrophoresed on a 10–12% SDS PAGE gel and prestained protein ladders (Thermofisher, Waltham, MA, USA) were loaded in parallel (as a molecular weight control) and then transferred to a PVDF membrane. After this, the membranes were blocked in 5% w/v skim milk in 0.1% TBS-T. Incubation of membrane with the respective primary antibodies anti-human Aβ-amyloid (1:200, Cat #28365, Santa Cruz Biotechnology, Dallas, TX, USA), Nicastrin (1:1000, Cat #D38F9, Cell Signaling Technology Inc., Waltham, MA, USA), Presenilin1 (1:1000, Cat #D39D1, Cell Signaling Technology Inc., MA, USA), Presenilin2 (1:1000, Cat #D30G9, Cell Signaling Technology Inc., Waltham, MA, USA), PEN-2 (1:1000, Cat #D6G9, Cell Signaling Technology Inc., Waltham, MA, USA), p-Tau^181^ (1:1000, Cat #D9F4G, Cell Signaling Technology Inc., Waltham, MA, USA), LOX-5 (1:1000, Cat # C49G1, Cell Signaling Technology, Inc. Waltham, MA, USA), LOX-12 (1:200, Cat #365194, Santa Cruz Biotechnology, Dallas, TX, USA), Akt (1:200, Cat #81434, Santa Cruz Biotechnology, Dallas, TX, USA), p-Akt (1:200, Cat #514032, Santa Cruz Biotechnology, Dallas, TX, USA), p53 (1:100, Cat #6243, Santa Cruz Biotechnology, Dallas, TX, USA), and anti-beta actin (1:1000, Cat #13E5, Cell Signaling Technology, Inc., Waltham, MA, USA) at 4 °C in 5% BSA was performed overnight. After overnight incubation, the membranes were washed three times with 0.1% TBS-T for 5 min each, followed by a reaction with the respective horseradish peroxidase-conjugated (HRP) secondary antibodies in 5% skim milk (NFM) for 1h at room temperature. This was followed by the washing of the membranes with 0.1% TBS-T thrice. For the detection of the bands, the enhanced chemiluminescent (ECL) detection reagent (FemtoLucent, GBiosciences, St. Louis, MO, USA) was used, and the densities of the bands were evaluated with My Image analysis (Thermo Fisher Scientific, Waltham, MA, USA). The graphs and statistical analyses were made with GraphPad prism (GraphPad Software, 5.0, San Diego, CA, USA).

### 2.10. Tissue Processing for Cryosectioning

The brains were removed carefully and stored in 4% PFA for 24 h at 4 °C. Next day after 24 h, the brains were washed in 1× PBS and transferred to a different percentage of sucrose solution gradient from 15 to 30%, prepared in 1× PBS at 4 °C, for proper cryoprotection. The brain samples were transferred from a low percentage sucrose solution to a higher percentage. Once the brain was found dipping at the bottom of the tube, samples were stored in 30% sucrose solution at 4 °C till cryo-sectioning.

The hippocampal tissue sections of 20 µm were obtained with a cryo-microtome and fixed with embedding medium OCT (Thermo Fischer Scientific, Waltham, MA, USA), then the tissue was fixed in a tissue holder and trimmed for proper sectioning. The tissue sections were gently adhered to Poly-L-lysine-coated frosted glass slides and were stored at 4 °C for immunofluorescence staining.

Cryo-sectioning of brain tissues was performed for immunofluorescence study by using the MICROM HM 550 (ThermoScientific, Waltham, MA, USA). The tissue freezing temperature was −20 °C for tissue sectioning.

### 2.11. Immunofluorescence Staining

The frosted glass slides were washed with PBS 0.01 mM for 8–10 min and the tissue section was permeabilized with 0.2% Triton X-100 at room temperature for 10–15 min, then it was blocked with blocking solution (1% Bovine Serum Albumin, 0.2% Triton X-100 in PBS) and incubated with the respective primary antibodies, anti-human Aβ-amyloid (1:200, Cat #28365, Santa Cruz Biotechnology), p-Tau^181^ (1:1000, Cat #D9F4G, Cell Signaling Technology Inc.), LOX-5 (1:1000, Cat # C49G1, Cell Signaling Technology, Inc.), LOX-12 (1:200, and Cat #365194, Santa Cruz Biotechnology). The mouse anti-NeuN (Ab104224, Abcam, Cambridge, UK) antibody was used to immunostain neurons. Then, the slides were washed with PBS and the respective fluorescent secondary antibodies were added (fluorescein isothiocyanate (FITC)-(green) or tetramethyl rhodamine (TRITC)-labeled secondary antibodies) for 2 h. For nuclear visualization, the slides were treated with 4,6-diamidino-2-phenylindole (DAPI) and mounted with a mounting media on coverslips. Images were captured using a confocal laser-scanning microscope (Leica, Wetzlar, Germany). For quantitative analysis the ImageJ software (version 1.46r) was used, and the graphs were generated with GraphPad Prism 5 software.

### 2.12. Quantification of LOX-5 and LOX-12 in Rat Serum by Surface Plasmon Resonance (SPR)

Quantification of LOX-5 and LOX-12 proteins in rat serum samples was performed by SPR using the BIAcore-3000 (Wipro GE Healthcare, Uppsala, Sweden), a biosensor-based system for real-time monitoring of specific interaction analysis. IgG primary rat monoclonal antibodies against LOX-5 (Cell Signaling Technology), and LOX-12 (Santa Cruz Biotechnology) were immobilized on the CM5 sensor chip using the amine coupling kit (Wipro GE Healthcare, Uppsala, Sweden). Rat serum samples were passed over the immobilized LOX-5 and LOX-12 antibodies and the RUs for each sample were recorded and the concentrations of LOX-5 and LOX-12 protein (ng/µL) were measured from the standard curve. The standard curves were prepared by passing different concentrations of purified recombinant LOX-5 and LOX-12 proteins over the immobilized antibody and corresponding resonance units (RU) noted [27] (Figure S1).

### 2.13. Statistical Analysis

Statistical analysis was performed using GraphPad Prism 5.0 (La Jolla, CA, USA). Bonferroni multiple comparison post hoc test was performed for the overall groups. Baseline comparison of quantitative measures between two groups was made using an independent *t*-test and for more than two groups a one-way ANOVA was used. *p* < 0.05 was considered as significant in all experimental results (*—*p* < 0.05, **—*p* < 0.001, ***—*p* < 0.0001, non-significant (ns)—*p* > 0.05).

## 3. Results

### 3.1. Synthesis and Mass Spectrometric Analysis of Aβ_25–35_ and YWCS Peptides

The synthesized Aβ_25–35_ and YWCS peptides mass analyses were confirmed by mass spectrometric analysis. The valve for the fraction collection was engaged as per the elution time of the peaks (Figure S2A,B). A fragmentation scan (MS2) was used for the analysis of the fragmentation pattern for the confirmation of desired peptide Aβ_25–35_. The first quadrupole (Q1) was fixed for 1060.4 Da and 60 V fragmentation energy was used. A slow infusion of the solution was carried out using a syringe pump fitted with the instrument. Figure S2C represents the MS2 spectra having product ions with the corresponding fragmentation sites. Aβ_25–35_ peptide MS spectra of fractions collected via flash chromatography is shown in Figure S2D. The purity of YWCS peptide was analyzed using high-performance liquid chromatography. The shadowed area shows the collected fraction for mass spectrometric analysis (Figure S2E). Precursor mass 558 [M + H] for the YWCS peptide was subjected to fragmentation using enhanced product ion (EPI) mode (Figure S2F).

### 3.2. AD-Rat Model and Intranasal Treatment of Peptide Inhibitor of LOX

The AD rat model was created by ICV injection of aggregated Aβ_25–35_. After 30 days of creating the AD rat model, the outcome of nasal treatment of the YWCS peptide for four weeks was demonstrated and monitored by the following experiments.

### 3.3. Behaviors Study

#### 3.3.1. Open Field (OF)

**Figure 1:**
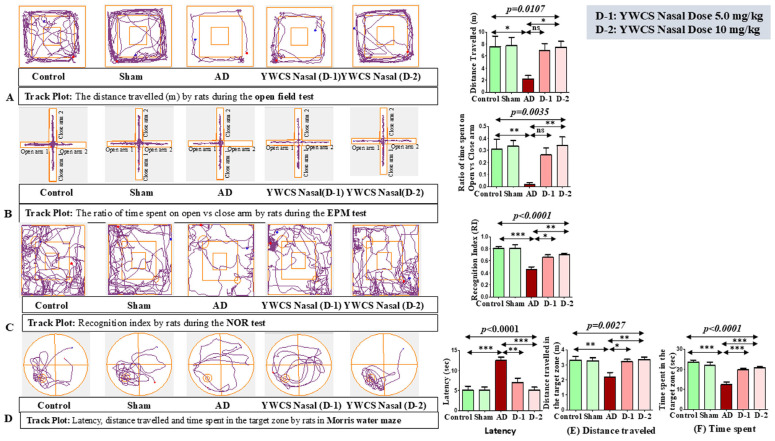
(**A**). Representative images of behavioral experiments with Bonferroni multiple comparison post hoc test. (**A**) Open field test: In the AD group, rats traveled less distance as compared to the control and sham groups, which upon giving the treatment of the peptide YWCS improved the activity of rats. (**B**) Elevated plus maze test: The ratio of time spent in the open vs. closed arm is less in AD rats compared to the control and sham groups due to fear and anxiety. The effect of the intranasal treatment of peptide YWCS was that it led to an improvement in the ratio of time spent in the open vs. closed arm. (**C**) Novel object recognition test: The recognition index declined in the AD group as compared to the control and sham groups and it increased in a dose-dependent manner after the intranasal treatment of peptide YWCS. (**D**) Morris water maze test: Latency to reach the platform, (**E**) distance traveled, and (**F**) time spent in AD rats were all higher compared to the control and sham groups. After the treatment of the peptide YWCS, the latency, distance traveled in the target zone, and time spent in the target zone declined significantly in both of the doses. (*—*p* < 0.05, **—*p* < 0.001, ***—*p* < 0.0001, non-significant (ns)—*p* > 0.05).

The aggregated Aβ_25–35_ induced anxiety-related behavior was evaluated in the open field arena, and it was found that the duration of movement was significantly declined in the AD group (*p* < 0.05) compared to control and sham group (Figure 1A). These parameters indicated that the ICV injection of aggregated Aβ_25–35_ induces anxiety and fear for exploring and movement in an open area. In AD rats, upon giving the treatment of the peptide YWCS intranasally at different doses, i.e., 5.0 mg/kg and 10 mg/kg, the duration of movement was significantly improved at a higher dose (10 mg/kg) (*p* < 0.05). However, no significant difference was observed at a lower dose (5.0 mg/kg). One-way analysis of variance showed significant inter-group difference (*p* = 0.0107), whilst a Bonferroni multiple comparison post hoc analysis was performed.

#### 3.3.2. Elevated Plus Maze (EPM)

This test is based on the unconditional fear response of rats to a challenge of height due to the development of anxiety. In AD rats, the ratio of time spent in the open vs. closed arm was significantly reduced compared to the control and sham groups (Figure 1B). AD rats spent more time in a closed arm compared to an open arm due to the development of fear and anxiety for exposure in the open area. In AD rats, upon giving the intranasal treatment of the peptide YWCS at different doses, i.e., 5.0 mg/kg and 10 mg/kg, the ratio of time spent in the open arm vs. time spent in the close arm was significantly improved at higher dose (10 mg/kg) (*p* < 0.05), i.e., they spent more time in the open arm. One-way analysis of variance showed a significance inter-group difference (*p* = 0.0035), and Bonferroni multiple comparison post hoc analysis was performed.

#### 3.3.3. Novel Object Recognition (NOR)

This test is based on the unconditional novelty preference of the rats. Generally, in normal behavior, rats spend more time exploring the novel object, but as the rats developed AD they spent a longer duration of time on the familiar object due to the impairment in the recognition memory. Due to the effect of aggregated Aβ_25–35_ ICV injection on the performance of the rats, the recognition index was declined in AD rats compared to the control and sham groups (Figure 1C). So, the AD rats seemed less interested in exploring objects. Interestingly, on intranasal treatment of the peptide YWCS, the recognition index recovered more at a higher dose (10 mg/kg). One-way analysis of variance showed significance inter-group difference (*p* < 0.0001), and it was followed by Bonferroni multiple comparison post hoc analysis.

#### 3.3.4. Morris Water Maze (MWM)

The Morris water maze behavioral test was used to analyzed the spatial memory and learning of the rats by evaluating (1) latency, (2) distance traveled in the target zone, and (3) time spent in the target zone. Latency was significantly increased in the AD group as compared to the control and sham groups (*p* < 0.0001). AD rats did several rounds in the water to reach the target zone. The treatment by the peptide YWCS intranasally at different doses, i.e., 5.0 mg/kg and 10 mg/kg, led to a significant decline in latency (Figure 1D).

The distance traveled (Figure 1E), (*p* = 0.0027) and time spent in the target zone (Figure 1F) (*p* < 0.0001) also significantly declined in the AD group as compared to the control and sham groups. On intranasal treatment with the peptide YWCS, the distance traveled and time spent in the target zone were both found to be significantly improved at both the doses (*p* < 0.05).

So, these parameters of behavioral test indicate that the ICV bilateral injection of aggregated Aβ_25–35_ induced memory impairment, and the treatment with the peptide YWCS improved the memory of the AD rats.

### 3.4. MRI Result

**Figure 2:**
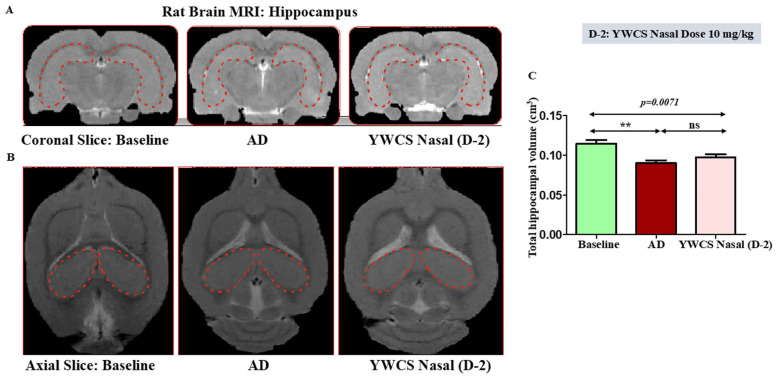
MRI images of AD rats exhibiting shrinkage of hippocampus as compared to baseline. After intranasal treatment of peptide YWCS at dose 10 mg/kg, no further shrinkage of the hippocampus was observed in AD rats. (**A**) Coronal Slice; (**B**) axial Slice; (**C**) bar diagram representing the changes in volume of hippocampus in 3 study groups, the red dotted shape represents the hippocampal region of the rat brain. (**—*p* < 0.001, non-significant (ns)—*p* > 0.05).

Sufficient resolution of the brain structures was provided by MRI scan to determine the volumetric changes in the coronal (Figure 2A) and axial slices (Figure 2B) region of the hippocampus and frontal cortex. We found that in the MRI scans for volumetric changes the AD group rats showed a significant (*p* < 0.05) reduction in hippocampal and frontal cortex volume compared to the baseline control group due to the shrinkage, which is a result of neurodegeneration. After intranasal treatment of peptide YWCS (10 mg/kg), no further shrinkage of the hippocampus and frontal cortex was observed, leading to the prevention of neurodegeneration. The overall significant changes in the volume of the hippocampus and frontal cortex (*p* = 0.0071) were observed across three groups of rats: the control group, the AD rat model group, and the AD model group treated with YWCS (Figure 2C).

### 3.5. Protein Expression Level Regulated by the Inhibitor YWCS by Immunoblotting

Protein expression levels in the hippocampus and frontal cortex were assessed across all study groups via immunoblot assay. β-actin was used as a loading control.

#### 3.5.1. Expression Analysis of Aβ-Amyloid, γ-Secretase Components, and Phosphorylation of p-Tau^181^ by Western Blot

**Figure 3:**
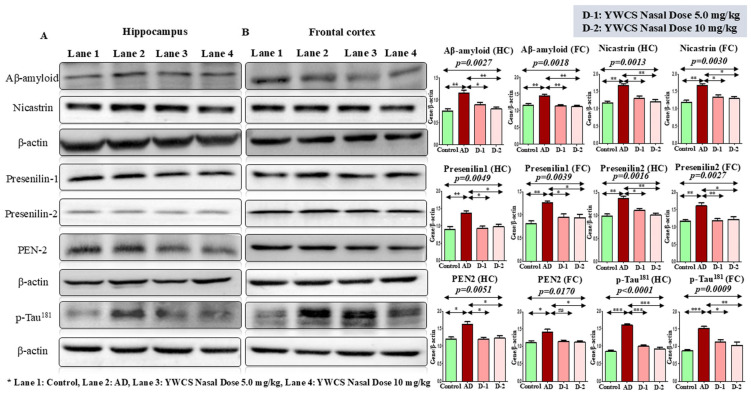
Immunoblots of the expression of Aβ-amyloid, γ-secretase components (Nicastrin, Presenilin-1, Presenilin-2, PEN-2), and p-Tau^181^ in (**A**) the hippocampus and (**B**) frontal cortex of a control, an AD, and an intranasal YWCS (5.0 mg/kg and 10 mg/kg) treated rat brain. These are normalized against β-actin. Comparative data are represented in bar diagram as mean ± SEM (*n* = 3). (*—*p* < 0.05, **—*p* < 0.001, ***—*p* < 0.0001, non-significant (ns)—*p* > 0.05).

During the pathogenesis of AD, γ-secretase leads to the formation of amyloid plaques and p-Tau^181^ forms neurofibrillary tangles. We found that the expression of Aβ-amyloid, γ-secretase components (Nicastrin, Presenilin-1, Presenilin-2, PEN-2), and p-Tau^181^ were significantly downregulated in the hippocampus and frontal cortex by the treatment of peptide YWCS at both the doses (i.e., 5.0 mg/kg and 10 mg/kg) (*p* < 0.05) (Figure 3A,B).

#### 3.5.2. Effect of YWCS on Expression of LOX-5 and LOX-12

**Figure 4:**
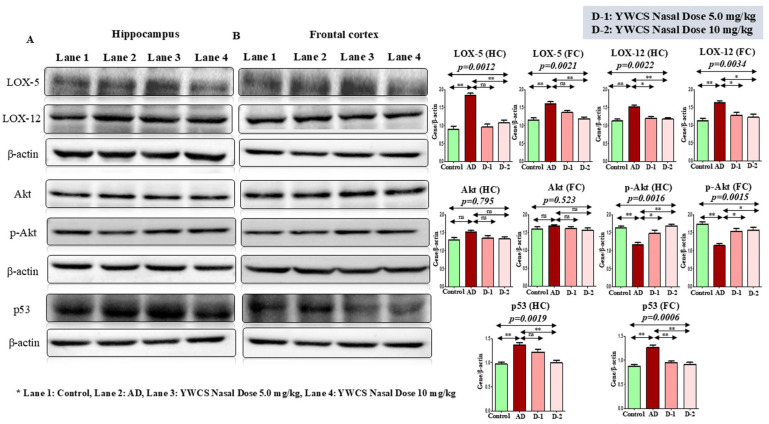
Immunoblots of the expression level of LOX-5, LOX-12, Akt, p-Akt, p53 in (**A**) the hippocampus and (**B**) the frontal cortex of a control, AD, and intranasal YWCS (5.0 mg/kg and 10 mg/kg) treated rat brain. These are normalized against β-actin. Comparative data are represented in the bar diagrams as mean ± SEM, (*n* = 3). (*—*p* < 0.05, **—*p* < 0.001, non-significant (ns)—*p* > 0.05).

The immunoblot results showed a significant (*p* < 0.05) increase in the expression of LOX-5 and LOX-12 proteins in the hippocampus and frontal cortex region in the AD group compared to the control group. After treatment of YWCS in the AD rat model, the expression of LOX-5 and LOX-12 were decreased significantly (*p* < 0.05) compared to the AD group in both the hippocampus and frontal cortex (Figure 4A,B).

#### 3.5.3. Effect of YWCS on Akt/p-Akt

##### AKT/p-AKT

The immunoblot results showed that the expression of Akt/p-Akt was downregulated in the AD group compared to the control (*p* < 0.05). After treatment of YWCS peptide the expression of Akt/p-Akt was upregulated significantly (*p* < 0.05) in both the hippocampus and frontal cortex. But, there was no change in the expression of Akt in both the hippocampus and frontal cortex. This result suggests that the inhibition of LOX-5 had no effect on Akt expression, but it induced the phosphorylation of Akt. So, a significant difference in the expression of Akt/p-Akt in the hippocampus and frontal cortex was observed (Figure 4).

#### 3.5.4. Effect of YWCS on the Expression of p53

It is also reported that LOX-5 regulates p53 activity. p53 is involved in a survival pathway mediated by Akt. The Western blotting experiment showed the expression of p53 was downregulated significantly (*p* < 0.05) at a higher dose (10 mg/kg) of peptide YWCS in the hippocampus and frontal cortex as compared to the AD group. The expression of p53 in the AD group was upregulated significantly (*p* < 0.05) compared to the control group in both the hippocampus and frontal cortex (Figure 4).

### 3.6. Immunofluorescence (IF) Assay

#### 3.6.1. Amyloid βeta Oligomers and p-Tau^181^ Diffusion and Accumulation in Rat Model

**Figure 5:**
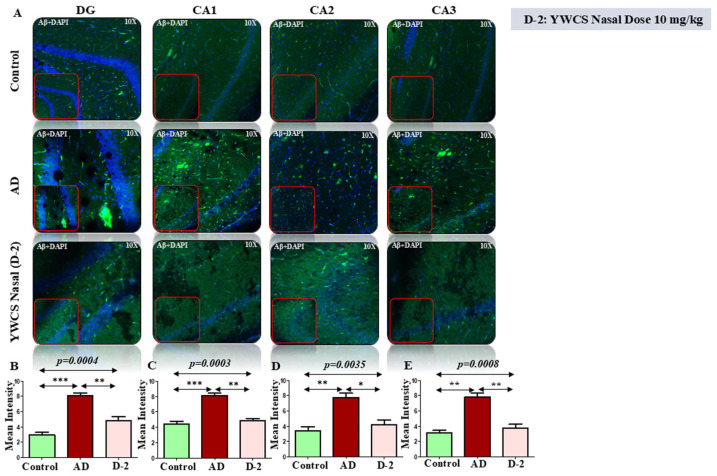
(**A**) The immunofluorescence of Aβ-amyloid in the DG, CA1, CA2, and CA3 regions of the hippocampus in an AD, control, and YWCS treated rat brain. Magnification 10X, scale bar 50 µm. (**B**–**E**) The comparative level of Aβ-amyloid in different regions is represented in the bar diagram (*n* = 3). Quantification of mean fluorescence intensity (MFI) with mean ± SEM. *—*p* < 0.05, **—*p* < 0.001, ***—*p* < 0.0001.

**Figure 6:**
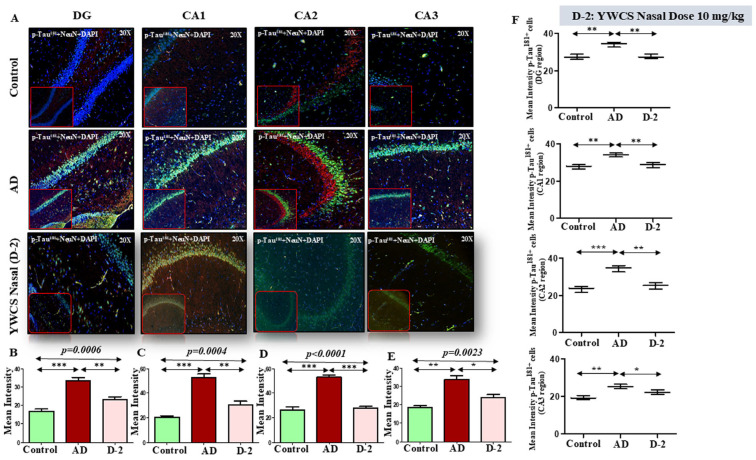
(**A**) Comparative difference in immunofluorescence staining of p-Tau^181^ in DG, CA1, CA2, and CA3 region of hippocampus in a control, AD, and YWCS nasal treated rat brain. (**B**–**E**) The quantification of mean fluorescence intensity (MFI) of p-Tau^181^ in different regions is represented in the bar diagrams. Magnification 20×, scale bar 50 µm. (**F**) The quantification of neuronal NeuN-positive p-Tau^181^ in different regions is represented in the scattered plot. (*—*p* < 0.05, **—*p* < 0.001, ***—*p* < 0.0001).

Amyloid βeta—The immunofluorescence assay of the DG, CA1, CA2, and CA3 parts of the hippocampal region of the brain showed high patches of Aβ-amyloid (FITC labeled, green) in the AD group as compared to the control group (*p* < 0.05). The treatment with peptide YWCS significantly reduced the effects of Aβ-amyloid in different parts of the hippocampus, i.e., DG (*p* = 0.0004), CA1 (*p* = 0.0003), CA2 (*p* = 0.0035), and CA3 (*p* = 0.0008) (Figure 5A–E).

p-Tau^181^—Robust accumulation of p-Tau^181^ (TRITC-labeled, red) was detected throughout the rat hippocampal region. The mean fluorescent intensity (MFI) of p-Tau^181^ in different parts of the hippocampal region was significantly increased in the AD model group as compared to the control group (*p* < 0.05). After treatment with peptide YWCS the mean fluorescent intensity of p-Tau^181^ in different parts of the hippocampus was significantly reduced, i.e., DG (*p* = 0.0006), CA1 (*p* = 0.0004), CA2 (*p* = 0.0001), and CA3 (*p* = 0.0023) (Figure 6A–E).

*Neuronal p-Tau^181^ expression*—Analysis of neuronal p-Tau^181^ revealed a consistent pattern of increased p-Tau^181^ intensity in the AD group compared to the control. On subsequent treatment with peptide YWCS, the p-Tau^181^ concentration significantly reduced in different regions of the hippocampus (Figure 6F).

#### 3.6.2. Lipoxygenases (LOX-5 and LOX-12)

##### LOX-5

**Figure 7:**
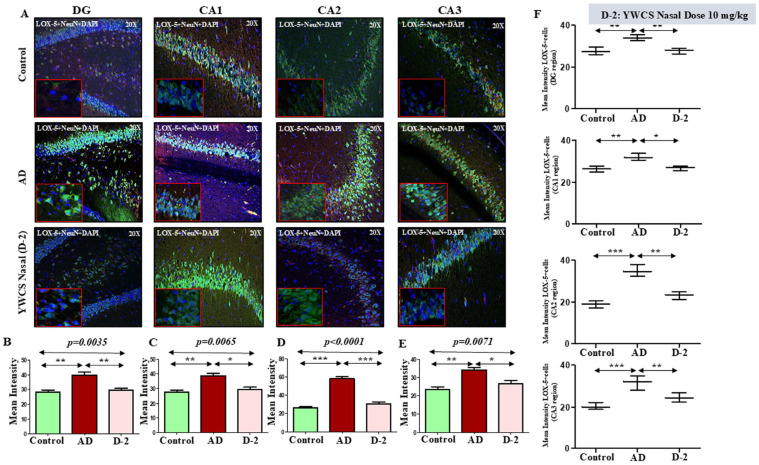
(**A**) Comparative difference in immunofluorescence staining of LOX-5 in the DG, CA1, CA2, and CA3 regions of the hippocampus in a control, AD, and YWCS nasal treated rat brain. (**B**–**E**) Quantification of the mean fluorescence intensity (MFI) of LOX-5 in different regions is represented in the bar diagrams. Magnification 20×, scale bar 50 µm. (**F**) Quantification of neuronal NeuN positive LOX-5 in different regions is represented in the scattered plot. *—*p* < 0.05, **—*p* < 0.001, ***—*p* < 0.0001.

LOX-5 expression appeared significantly increased in the AD group in different subfields of the hippocampus compared to the control group (*p* < 0.05). Treatment with peptide YWCS significantly reduced the mean fluorescence intensity of LOX-5 compared with AD group in different subfields of the hippocampus, i.e., DG (*p* = 0.0035), CA1 (*p* = 0.0065), CA2 (*p* < 0.0001), and CA3 (*p* = 0.0071) (Figure 7A–E).

*Neuronal LOX-5 expression*—Analysis of neuronal LOX-5 revealed the consistent pattern of increased LOX-5 intensity in the AD group compared to the control. On subsequent treatment with peptide YWCS, the LOX-5 concentration significantly reduced in different regions of the hippocampus (Figure 7F).

##### LOX-12

**Figure 8:**
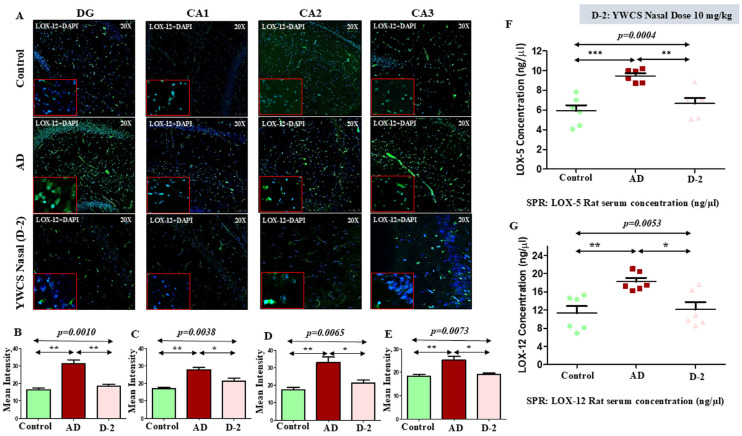
(**A**) Quantification of mean fluorescence intensity (MFI) of LOX-12 in DG, CA1, CA2, and CA3 in the hippocampus in a control, AD, and YWCS treated rat brain by immunofluorescence. Magnification 20X, scale bar 50 µm. (**B**–**E**) The comparative level of LOX-12 in different regions is represented in the bar diagrams (*n* = 3). MFI with mean ± SEM. The expression level of serum (**F**) LOX-5 and (**G**) LOX-12 in control, AD, and YWCS treated rats by SPR. *—*p* < 0.05, **—*p* < 0.001, ***—*p* < 0.0001.

The mean fluorescence intensity of LOX-12 in different parts of the hippocampus was significantly higher in the AD group compared to the control group (*p* < 0.05). The treatment with peptide YWCS on the AD rats significantly declined the LOX-12 mean fluorescence intensity in different subfields of the hippocampus, i.e., DG (*p* = 0.0010), CA1 (*p* = 0.0038), CA2 (*p* = 0.0065), and CA3 (*p* = 0.0073) (Figure 8A–E).

### 3.7. Quantitative Analysis of Rat Serum LOX-5 and LOX-12 by SPR

The evaluation of LOX-5 concentration by SPR showed a significant increase in the concentration of LOX-5 in the serum of an AD model rat compared to the control group (control 5.89 ng/µL, AD 9.46 ng/µL, and YWCS nasal (D-2) 6.65 ng/µL). The AD model group upon being given the treatment with YWCS peptide showed a significant reduction in the concentration of LOX-5 compared with the serum of the AD rat model group (Figure 8F).

Similarly, in the AD group LOX-12 concentration was significantly increased as compared to the control (control 11.29 ng/µL, AD 18.22 ng/µL, and YWCS nasal (D-2) 12.11 ng/µL). The concentration of LOX-12 was significantly declined in the YWCS treated group as compared to the AD group (Figure 8G).

## 4. Discussion

Memory decline is often seen as a typical issue of aging. However, when memory loss progresses to AD, it transforms into a major health concern for older adults. The defining features of AD in the brain are the presence of senile plaques, which are built up from Aβ amyloid peptide outside nerve cells, and neurofibrillary tangles (NFTs) forming inside these cells. These changes are particularly concentrated in the brain cortex region, mainly in the hippocampus, which is crucial for memory and cognition. Beyond these characteristic lesions, significant activation of inflammatory pathways also plays a role in the disease process. There is epidemiologic evidence linking inflammatory drug use to reduced AD risk. LOX and COX are found to be overexpressed in AD, which enhances several cytokines and damages neurons [28]. This study focused on role of LOX in AD pathogenesis. LOX-5 protein levels are particularly enriched in the frontal cortex and hippocampus [29], which are known to be vulnerable in neurodegeneration, and in AD in particular [30,31]. The metabolic products of LOX-5, leukotrienes, initiate immune cell chemotaxis and play critical roles in inflammatory pathways [32]. It has been reported that zafirlukast can reduce oxidative stress and protect against neurotoxicity in an AD rat model by inhibition of the release of LOX metabolites, which are known to contribute to inflammation and oxidative damage in the brain [33]. LOX-5 was found to be increased in post-mortem AD brains [34,35]. Knockout of the *LOX-5* gene reduces the activity of γ-secretase complexes, lowering steady-state expression of nicastrin, presenilin-1 (PS1), APH-1, and PEN-2 proteins without affecting either APP, β-secretase, or α-secretase pathways [36,37], thereby improving learning and memory performance in a rat model over baseline. Conversely, over-expression of LOX-5 is associated with cognitive impairment [36]. A specific inhibitor, zileuton, attenuates ischemic brain damage by inhibiting inflammatory reactions through the activation of the PI3K/Akt signaling pathway. Because of structural similarities involving the active site of LOX-12 and LOX-5 [12,38], peptide YWCS was designed by in silico modeling and appeared to be an effective inhibitor of both LOXs [11,27], and thus it behaves like a “dual inhibitor” which showed anti-breast cancer activity in breast-cancer cell lines [12] and also anti-AD. There is little evidence that cancer drugs can prevent AD and PD [39]. The anti-cancer drug bexarotene prevents nucleation of toxic Aβ_25–35_ and prevents AD [40]. The Aβ_25–35_ peptide induces neuronal cell death by exerting neurotoxic effects in AD by various mechanisms [41]. Harmine-1, a naturally occurring molecule, has demonstrated potential therapeutic benefits by exhibiting anti-inflammatory and anti-AD properties by inhibiting the enzyme LOX-5 and acetylcholinesterase, thus preventing the breakdown of acetylcholine in the brain [42]. Notably, Harmine-1 has also shown anti-cancer activity against breast cancer cell lines. Similarly, the compound YWCS has displayed both anti-cancer and neuroprotective effects by reducing the excessive production of γ-secretase and the hyperphosphorylation of tau protein. Rats are a suitable species for biomedical research due to their anatomical, physiological, and genetic similarity to humans [43]. Aβ-amyloid was injected into rat brains to create an AD rat model, as reported earlier [44]. Social withdrawal, depression, anxiety, and memory loss are common behavioral changes in AD patients, and these were also reflected in our experimental AD rat model using different behavioral tools. NOR testing was used to evaluate recognition memory and the normally spontaneous tendency of rodents to spend more time exploring a novel object than a familiar one. Due to the decline in short-term memory, AD rats could not spend time with novel objects. However, rats after treatment with peptide delivered by nasal spray improved significantly. Open field tests were used to define the anxiety of rats, and the total distance traveled by the animals, which both measure anxiety-related behaviors and memory. The AD rats in our experiment were constrained in movement and traveled shorter distances compared to normal rats, which were both significantly improved after peptide treatment delivered intranasally. Similarly, we used the elevated plus maze test, the most extensively employed assessment for measuring anxiety depressive disorders. In our experiments, AD rats spent most of the time in closed arms rather than open arms due to anxiety and were confined in closed areas. In contrast, the peptide treatment of rats significantly improved this behavioral nature. The Morris water maze experiment, developed by Richard Morris to measure strong and reliable spatial memory, correlates with hippocampal synaptic plasticity [45]. A significantly higher latency rate in AD rats compared to control animals was observed due to the decline in memory because of AD. This is due to the prolonged time taken to locate the escape platform, which was improved in treated rats with better cognitive function due to a significant decrease in latency. These all-behavioral changes in AD rats mimics the memory loss and other cognitive impairments in AD patients. After intranasal delivery of YWCS, rats exhibited significant improvements in cognitive function in the AD model rats. The hippocampus and frontal cortex mainly control memory functions of the brain and shrinkage of these regions influences memory loss and cognitive function [46,47]. Nasal treatment with YWCS prevented further shrinkage of the hippocampus and frontal cortex of rats. Hence, it can be assumed that this peptide inhibitor of LOX prevented the glial cells and thus prevented further accumulation of plaques and tau tangles. LOX promotes the generation of Aβ by inducing the γ-secretase complex and regulates tau protein metabolism by altering its phosphorylation status at particular sites through cyclin-dependent kinase-5 (cdk-5). The expression of Aβ-amyloid, γ-secretase components, and p-Tau^181^ were found to be overexpressed in AD rats in our experiments and found to be downregulated after intranasal treatment with the peptide YWCS. Several lines of evidence indicate that during oxidative stress, Zn is released, which activates LOX-12, thereby increased levels of LOX-12 are found in the frontal temporal cortex in post-mortem brains [48]. In our study, LOX-5 and LOX-12 were found to be overexpressed in the hippocampus and frontal cortex in the brains of AD rats compared to healthy rats. In our previous study, we reported that inhibition of LOX by YWCS in SH-SY5Y cells upregulates the phosphorylation of Akt, and the same reciprocal was observed in AD rats treated with YWCS in the present study. Increased phosphorylation of Akt prevents the formation of aggregated Aβ amyloid in the same rats. Brain oxidative stress in the early onset of AD was found to be associated with high expression levels of LOX in the cortex and hippocampus of post-mortem brains [48]. The immunofluorescence study of the post-mortem brains of AD rats in our study revealed high expression of LOX in the hippocampus, mainly in the DG and CA2 regions, and which were found to be low after treatment with the inhibitor YWCS. The DG region encodes memory in the brain and the CA2 region [49] is paramount to the social recognition memory process as well as maintaining the memory of familiar and novel objects. Hence, it can be speculated that due to the over-production of inflammatory protein LOX in DG and CA2 regions, it initiates the formation of Aβ-amyloid aggregation and damages neurons, which affects memory function in rats. LOX peptide inhibitor treatment delivered by nasal spray reduced LOX expression as well as Aβ-amyloid aggregation. The nasal spray treatment was effective as the CA2 region responded to olfactory signals. Immunofluorescence assays involving the hippocampus region in AD rats showed high expression of Aβ-amyloid as well as p-Tau^181^ along with high expression of LOX protein, while no alterations involving these protein levels were observed in normal rats. DG and CA2 regions showed significant improvement in our YWCS-treated model in terms of the expression of Aβ-amyloid and p-Tau^181^. Earlier, it was shown that knockout or inhibition of LOX-5 restores hippocampal long-term potentiation and improves cognitive deficits, synaptic loss, and neuropathology in a transgenic mouse model of AD [34]. Drugs for any brain disorder delivered by oral or intravenous routes do not readily pass the blood–brain barrier (BBB) and some are degraded by metabolic processes [50,51]. Hence, the amount of drug which actually enters the brain is insufficient for therapeutic effects. Nasal administration of drugs has the advantage of avoiding the BBB, as well as metabolic circulation and that the drugs directly reach the olfactory nerve [51]. The respiratory region is part of several senses of cognition which, along with the olfactory region, take part in the intranasal transmission of drugs directly to the brain, tailoring formulations for intranasal delivery [52]. Protein ligand interactions based on small-molecule therapeutic agents have attracted interest due to their specificity, avoiding interactions with larger areas to avoid side-effects. The peptide YWCS was found to be a specific inhibitor of LOX without any toxic side-effects. A decline in LOX expression in brain tissue is found to be reflected in improvements in cognitive behaviors in AD rats in response to Y-maze tests, NOR paradigms, and open field tests in both working memory and MWM for spatial and learning memory. Significant difference in the serum LOX levels in rats were observed to be lower in normal rats compared to AD rats, and this was downregulated by treatment with YWCS.

The present observations of elevated LOX levels in an in vivo AD rat model, along with the prevention of AD pathological symptoms by YWCS treatment, corroborates our previous research findings of high LOX levels in the blood of AD patients compared to healthy elderly individuals and shows the neuroprotective effects of the YWCS peptide in AD cell line models. It can be summarized from the present study that increased Aβ-amyloid and hyperphosphorylated tau levels were associated with significant cognitive impairments correlated with overexpression of LOX in the hippocampus and frontal cortex of experimental AD model rats. Remarkably, intranasal administration of YWCS peptide, a specific inhibitor of LOX, demonstrated a significant downregulation of Aβ and p-Tau181 levels, preventing further brain shrinkage and markedly improving cognitive functions.

## 5. Conclusions

LOX plays a significant role in neuroinflammation, a key pathological process in AD. Elevated LOX can trigger neuroinflammation by inducing neurotoxicity via increasing amyloid plaque deposition and tau tangles in the brain. In this study, we found aggregated Aβ_25–35_ creates AD pathogenesis in terms of behavioral and anxiety functions by increasing Aβ-amyloid and p-Tau^181^ in AD model rats in the hippocampus and frontal cortex, with a shrinkage in brain volume. Interestingly, the LOX inhibitor YWCS peptide improved cognitive dysfunction and prevented further shrinkage of the brain by reducing levels of LOX and subsequently Aβ-amyloid and p-Tau^181^. This suggests that inhibition of LOX may prevent the neuroinflammation and poor cognitive dysfunction related to AD pathogenesis, with significant translational potential.

YWCS may emerge as a highly promising therapeutic candidate for AD. This highlights the crucial steps required in future studies for translating the findings from bench to bedside.

## Data Availability

All the data are available in the manuscript.

## Supplementary Materials

The following supporting information can be downloaded at: https://www.mdpi.com/article/10.3390/cells14130957/s1, Figure S1 A. Immobilization sensorgram of anti-human LOX-5 antibody on CM5 chip and standard curve B. Immobilization sensorgram of anti-human LOX-12 antibody on CM5 chip and standard curve. Figure S2 A. Chromatogram at 254 nm for blank injection B. Chromatogram at 254 nm for peptide sample injection. Note—peaks mentioned in shadows were collected by fraction collector C. MS2 fragmentation pattern of collected fraction. Dashed red lines show corresponding fragmentation sites D. Aβ_25-35_ peptide- MS spectra of fractions collected via Flash chromatography E. Peptide (YWCS) purity analysis using high performance liquid chromatography. The shadowed area shows the collected fraction for mass spectrometric analysis F. Enhanced product ion (EPI) scan for the fraction collected. Precursor mass 558 [M+H] for YWCS peptide was subjected to fragmentation using EPI mode. Note—The alphabets show the exact fragmentation site on the YWCS structure.

## Abbreviations

AD, Alzheimer’s disease; Aβ, β-amyloid; APP, amyloid precursor protein; ECL, enhanced chemiluminescent detection reagent; EPM, elevated plus maze; FITC, fluorescein isothiocyanate; ICV, intracerebroventricular; IF, immunofluorescence; LOX, Lipoxygenase; MFI, mean fluorescent intensity; MWM, Morris water maze; NOR, novel object recognition; RI, recognition index; RU, response unit; TRITC, tetramethyl rhodamine.
