# Supplementary material for: Anti-Inflammatory Peptide Prevents Aβ25–35-Induced Inflammation in Rats via Lipoxygenase Inhibition
Source: Cells. 2025 Jun 23;14(13):957. doi: 10.3390/cells14130957 (PMC12249324; doi:10.3390/cells14130957)
Supplement: Supplementary file 1 [file cells-14-00957-s001.zip › cells-3673694-supplementary.pdf]

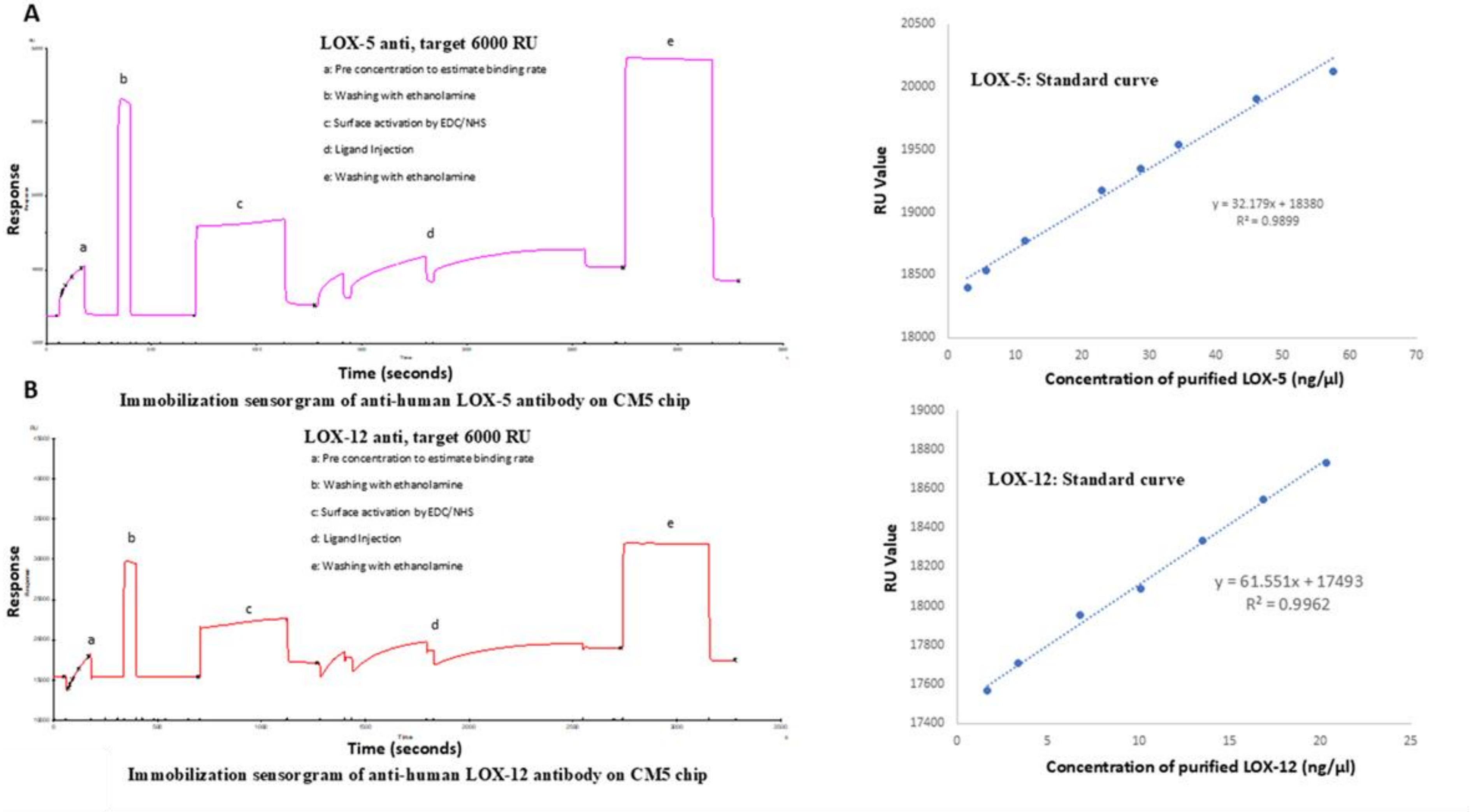

Figure S-1 A. Immobilization sensorgram of anti-human LOX-5 antibody on CM5 chip and standard curve B. Immobilization sensorgram of anti-human LOX-12 antibody on CM5 chip and standard curve

A

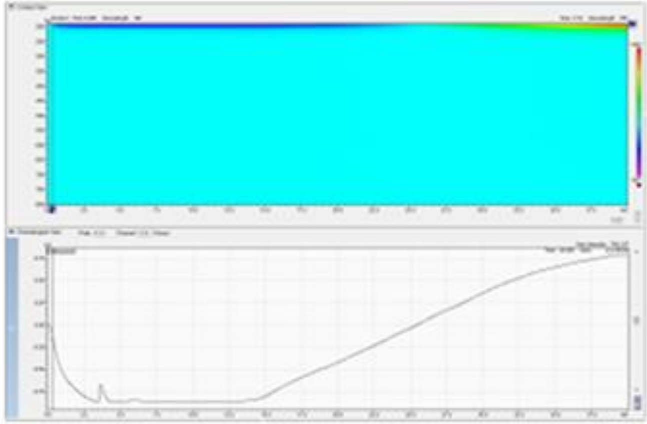

Chromatogram at 254 nm for blank injection

C

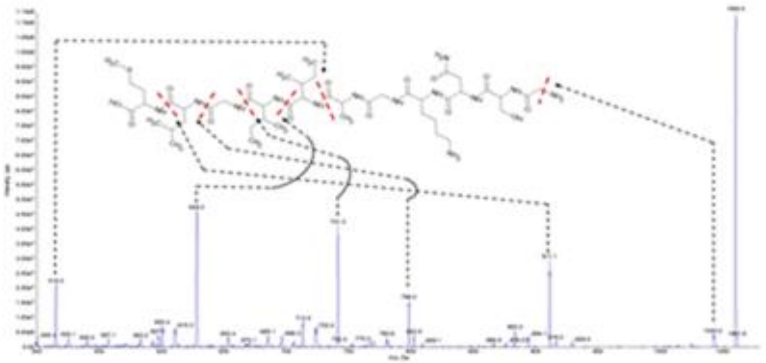

MS2 fragmentation pattern of collected fraction. Dashed red lines show corresponding fragmentation sites

E

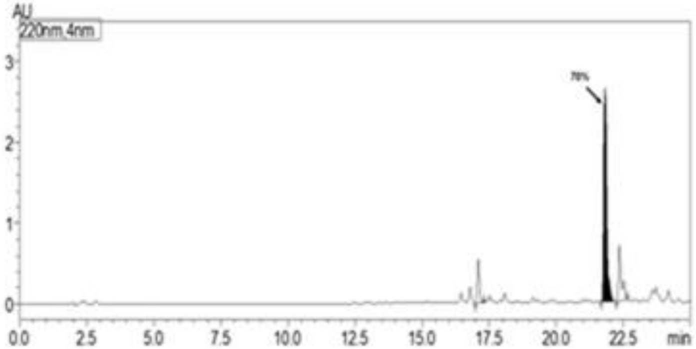

YWCS Peptide purity analysis using high performance liquid chromatography. The shadowed area shows the collected fraction for mass spectrometric analysis

B

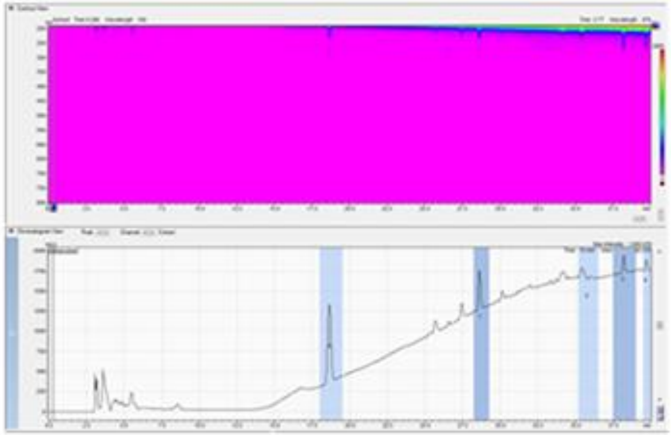

Chromatogram at 254 nm for peptide sample injection. Note – peaks mentioned in shadows were collected by fraction collector

D

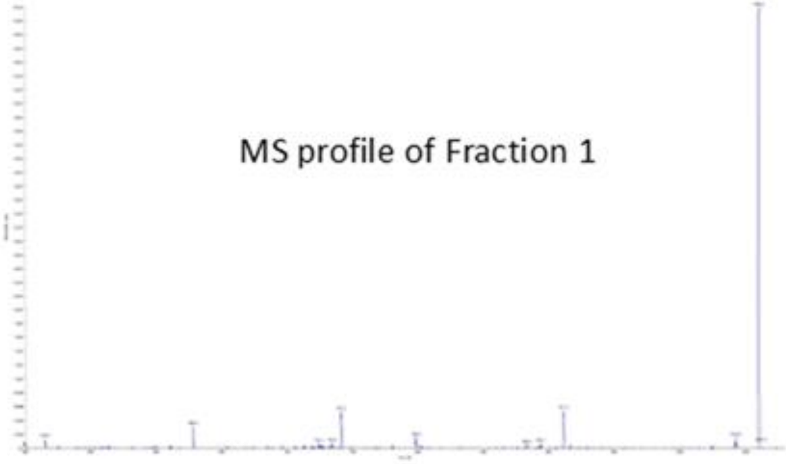

Aβ_{25-35} peptide- MS spectra of fractions collected via Flash chromatography

F

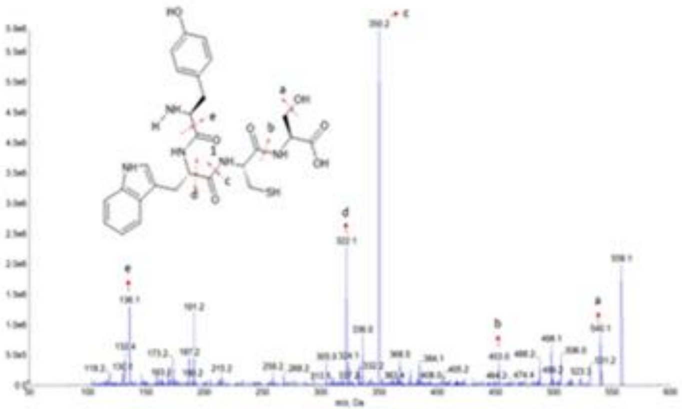

Enhanced product ion (EPI) scan for the fraction collected. Precursor mass 558 [M+H] for YWCS peptide was subjected to fragmentation using EPI mode. Note – The alphabets show the exact fragmentation site on the YWCS structure

Figure S2 A. Chromatogram at 254 nm for blank injection B. Chromatogram at 254 nm for peptide sample injection. Note – peaks mentioned in shadows were collected by fraction collector C. MS2 fragmentation pattern of collected fraction. Dashed red lines show corresponding fragmentation sites D. Aβ25-35 peptide- MS spectra of fractions collected via Flash chromatography E. Peptide (YWCS) purity analysis using high performance liquid chromatography. The shadowed area shows the collected fraction for mass spectrometric analysis F. Enhanced product ion (EPI) scan for the fraction collected. Precursor mass 558 [M+H] for YWCS peptide was subjected to fragmentation using EPI mode. Note – The alphabets show the exact fragmentation site on the YWCS
